# Validating Internal Control Genes for the Accurate Normalization of qPCR Expression Analysis of the Novel Model Plant *Setaria viridis*

**DOI:** 10.1371/journal.pone.0135006

**Published:** 2015-08-06

**Authors:** Julia Lambret-Frotté, Leandro C. S. de Almeida, Stéfanie M. de Moura, Flavio L. F. Souza, Francisco S. Linhares, Marcio Alves-Ferreira

**Affiliations:** Department of Genetics, Universidade Federal do Rio de Janeiro, UFRJ, Rio de Janeiro, Brazil; National Institute of Plant Genome Research, INDIA

## Abstract

Employing reference genes to normalize the data generated with quantitative PCR (qPCR) can increase the accuracy and reliability of this method. Previous results have shown that no single housekeeping gene can be universally applied to all experiments. Thus, the identification of a suitable reference gene represents a critical step of any qPCR analysis. *Setaria viridis* has recently been proposed as a model system for the study of Panicoid grasses, a crop family of major agronomic importance. Therefore, this paper aims to identify suitable *S*. *viridis* reference genes that can enhance the analysis of gene expression in this novel model plant. The first aim of this study was the identification of a suitable RNA extraction method that could retrieve a high quality and yield of RNA. After this, two distinct algorithms were used to assess the gene expression of fifteen different candidate genes in eighteen different samples, which were divided into two major datasets, the developmental and the leaf gradient. The best-ranked pair of reference genes from the developmental dataset included genes that encoded a phosphoglucomutase and a folylpolyglutamate synthase; genes that encoded a cullin and the same phosphoglucomutase as above were the most stable genes in the leaf gradient dataset. Additionally, the expression pattern of two target genes, a SvAP3/PI MADS-box transcription factor and the carbon-fixation enzyme PEPC, were assessed to illustrate the reliability of the chosen reference genes. This study has shown that novel reference genes may perform better than traditional housekeeping genes, a phenomenon which has been previously reported. These results illustrate the importance of carefully validating reference gene candidates for each experimental set before employing them as universal standards. Additionally, the robustness of the expression of the target genes may increase the utility of *S*. *viridis* as a model for Panicoid grasses.

## Introduction

Science is now experiencing the era of genomics and transcriptomics [1]. Over the past few years, the use of next-generation sequencing (NGS) has rapidly increased the amount of data that can be generated and has thus transformed plant sciences [2]. However, the widespread adoption of this technology has been limited both by its high cost and by the non-trivial level of computational analysis that is required to evaluate the large quantity of DNA and/or RNA sequences that are produced; particularly for organisms that have complex genomes (e.g., sugarcane) [1,3].

The plasticity that plants exhibit when exposed to different environmental circumstances has raised interest into their regulatory networks and patterns of gene expression; this is especially true with regard to the responses of economically relevant crops to variations in the planting field [4–6]. In this context, real-time quantitative polymerase chain reaction (qPCR) represents an attractive means of evaluating the expression profiles of genes of interest within a large set of biological samples. Furthermore, qPCR analysis has become the method of choice for validating the transcriptome data and to facilitate in-depth expression studies of smaller sets of genes, including studies of alternative splicing, verification of microarray expression results and molecular diagnostics [7,8]

qPCR is a straightforward and well-established technique that is primarily based on the exponential incorporation of fluorescent molecules into genetic material [9]. It is a robust method for precisely quantifying changes in gene expression over a wide dynamic range [10]. There are two processes that are primarily required to successfully produce reliable results using qPCR, including both RNA extraction/purification [11] and data expression normalization using reliable reference genes [12]. The purpose of normalization is to correct for non-specific variability, such as differences in RNA quantity and quality and the non-linear efficiency of reverse transcription and PCR amplification [13]. Thus, measurements of gene expression that have not been sufficiently normalized may lead to biased results and erroneous interpretation [10,13–15].

*Setaria viridis* is a species of grass that is a member of the Panicoid family, which is the most economically important family of grass species, and includes maize, sorghum and sugarcane [16]. Each of the above listed members of the Panicoid family undergoes C4 photosynthesis of carbon capture and fixation. They are also morphologically characterized as having Kranz anatomy, such that bundle-sheath (BS) and mesophyll (M) cells arranged in concentric circles around the vascular bundles of the leaves [17]. C4 photosynthesis can be subdivided into three main subtypes, which are biochemically distinct [18]. In the case of *S*. *viridis*, atmospheric CO_2_ is first fixed in the M cytoplasm by PEPC (phosphoenol-pyruvate carboxylase), and after chemical modifications it is transformed into a four-carbon compound (malate). The malate is then transported into BS chloroplasts where the decarboxylation step is performed mainly by the NADP-dependent malic enzyme (NADP-ME), leading to the release of CO_2_ close to the active site of the RuBisCO enzyme [19]. In C4 plants, RuBisCO is confined to BS cells, and this partitioning of carbon assimilation and carbon fixation steps greatly reduces the losses caused by photorespiration [20]. Such an arrangement enables these plants to more efficiently use water and nitrogen, especially in hot and dry environments [21].

Due to the importance of C4 syndrome in biomass production [22], major efforts are being made to understand the developmental changes that have led to the multiple instances of C4 photosynthesis over the course of plant evolution [19,21,23,24]. In this regard, *S*. *viridis* has been proposed as a genetic model for C4 photosynthesis studies in grasses [25] and many genetic tools have been created to validate its use [25–29]. Considering the important role of qPCR in gene expression analysis, in this study we intend to present candidate genes that can be employed to accurately normalize overall gene expression during qPCR analysis of *S*. *viridis* samples. We have analyzed the expression levels of 15 candidate genes over the course of the *S*. *viridis* life cycle in different tissues. We also exploited the leaf developmental gradient [30] to provide robustness for further gene expression analysis during later events of leaf differentiation.

## Materials and Methods

### Plant growth

*S*. *viridis* (accession A10.1) seeds were placed on filter paper, covered with water and incubated for 48 h at 4°C to promote dormancy break. Following this treatment, the seeds were transferred into a soil mixture and placed in a greenhouse at Universidade Federal do Rio de Janeiro (Rio de Janeiro, Brazil), which provided a natural photoperiod and temperatures ranging from 30–35°C.

**Fig 1:**
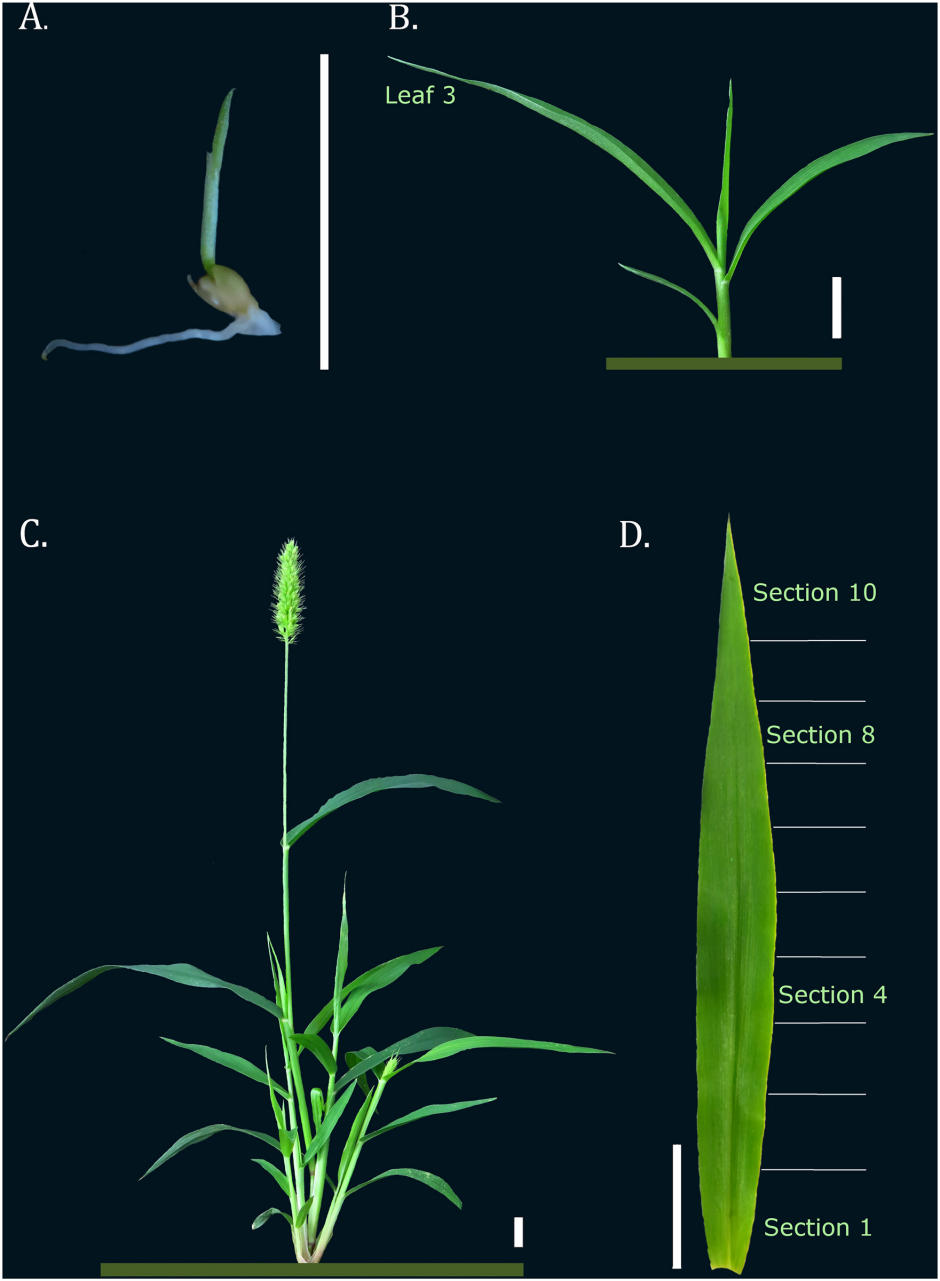
Illustrative figure of each *S*. *viridis* tissue and developmental stage that was sampled. A. Seedling stage (3–5 DAS); B. Young *S*. *viridis*, with third leaf fully expanded; C. Mature *S*. *viridis*; D. The third leaf 0.5 cm sections that comprised the leaf gradient analysis. Bar corresponds to 1.0 cm.

The seedlings were harvested 3–5 days after sowing, after the coleoptile had appeared. Young plants were collected as soon as the third leaf was completely expanded; in each, the root was separated from the shoot, and the third leaf comprised a different sample. Based on the developmental gradient of the first C4 leaf, the third leaf of each young plant was sectioned into 0.5 cm pieces transversally from the ligule; each section was individually collected and later pooled with equivalent section samples. Mature plants were harvested after they reached reproductive maturity. Their roots were separated from their shoots, inflorescences and floral axes, and each of these became an individual sample (Fig 1).

Three biological samples were evaluated and each comprised a pool of at least five different plants from the developmental set and of 20 plants from the leaf gradient set. The samples were immediately frozen on liquid nitrogen and kept at -80°C until they were used for RNA extractions.

### Candidate genes and primer design

Candidate genes for qPCR normalization were selected based on previous studies conducted on maize [31] and rice [32] (Table 1). The nucleotide sequences of the 15 candidate genes, and an additional two target genes, were BLAST searched against the Phytozome database (http://phytozome.jgi.doe.gov/) to identify homologues in the *S*. *italica* genome (v2.1), which is a close relative of *S*. *viridis*. Sequences with e-values lower than 10E-40 were selected for primer design. The homology was confirmed based on alignment with the transcriptome sequencing of *S*. *viridis* leaves (Table 1; personal communication with Todd Mockler). The *S*. *viridis* sequences are not available on public DNA databases, therefore the accession numbers are not shown on Table 1.

**Table 1:** Candidate genes and their primer sequences that were selected for evaluation of expression stability using qPCR analysis on *S*. *viridis* tissues, and the sequences of two genes of interest.

Phytozome ID		BLAST e-value (*S*. *italica*)	*Setaria viridis*		Biological Function	Primer Sequence (Fw/Rv)	cDNA Amplicon	gDNA Amplicon
*Setaria italica*	*Zea mays/ Oryza sativa*		Alignment Score^1^	Identity^1^ (%)				
***Reference Genes Candidates***								
Si018608	GRMZM2G027378	1,20E-56	98.16	100	Ubiquitin-conjugating enzyme	CAGTGAGCTATGGAATGGATGA/ GACGCATGTCATGTTGATTCTT	185 bp	564 bp
Si025395	GRMZM2G080603	4,00E-50	NA	NA	Glycine-rich RNA-binding protein	GGAGATGAGGATAGCTCTGACG/ TGTCTAGTGTCTTCGTCGTCGT	122 bp	122 bp
Si018607	GRMZM2G102471	1,40E-55	NA	NA	Ubiquitin-protein ligase	CCTCCAGACTATCCATTCAAGC/ CTCCACTGCTCCTTGAGAATGT	113 bp	240 bp
Si035045	GRMZM2G393334	6,60E-86	97.16	99	Folylpolyglutamate synthase	AAGTTTTTGCGTCACTTCCACT/ CAAATTTCTGCCCTCGCTAAT	139 bp	500 bp
Si000245	GRMZM2G425377	1,90E-92	99.42	100	WD40 repeat-containing protein/ Leunig	GGACATGATGGTGATTTTGTGA/ TTCTTCTCCCTTATGTCCCAAA	140 bp	222 bp
Si021145	GRMZM2G164418	0	98.74	99	Ubiquitin carboxyl-terminal hydrolase	ATTCCCCACAATTATCGACTGT/ TCAAAGTCGTACCACCCTTTTT	113 bp	746 bp
Si021373	GRMZM2G166694	1,30E-121	99.07	99	Cullin	TATGGGTCATCAACAGCTTGTC/ GTAGTCCCTCGTGATGAGATCC	112 bp	534 bp
Si034613	GRMZM2G109383	9,10E-66	99.09	99	Phosphoglucomutase	ACGAGAAGGATTCATCCAAGAC/ CGTGTACTCTTGCATCTTGGAG	98 bp	195 bp
Si017354	GRMZM2G018103	9,10E-127	99.19	99	Serine incorporator	GACTGCTAACCACCATCCTTTC/ CTTCTTGTTCATCAGCCTTGCT	138 bp	223 bp
Si022372	GRMZM2G126010	0	92.53	100	Actin	GGACATTAAAGAGAAACTCGCGTA/ ACCTTTCTGATCCAATGGTGATA	134 bp	134 bp
Si035654	GRMZM2G152466	0	93.3	99	Tubulin	CTCGCCCTCGTCGAACT/ ACAAGTTTGACCTCATGTACGC	152 bp	152 bp
Si030042	LOC_Os03g08010	0	91.37	84	Elongation factor	TTCTCGGAGCTGCTGACC/ CGGGACCATCTTGACGAG	105 bp	105 bp
Si014034	LOC_Os08g03290	4,70E-47	98.42	99	GAPDH	GGATTTGGTTTCCACTGACTTC/ CTACTGGGTCTTGAACATGTGG	172 bp	325 bp
Si002651	LOC_Os01g05490	1,20E-46	97.61	99	Triosephosphate isomerase	ACTGCTGCAAACTGCAAAGAG/ GTTGATGATGTCGATGAACTCAG	99 bp	238 bp
Si003209	LOC_Os01g22490	0	93.86	99	Ribosomal protein	TATGTTGGGGAGAAGGAAAATG/ CATCACCATAAGAGATGCAGGA	110 bp	110 bp
***Target Genes***								
Si002929	GRMZM2G110153	3,50E-84	NA	NA	MADS-box AP3/PI	AACAAGCTGCTGTCCTTTAAGC/ CGGAAGGTTATTGGCATCTG	122 bp	693 bp
Si000160	GRMZM2G083841	0	96.35	100	PEPC	CTAACATCCCGGAAGACAAGAC/ GAGCTTGACGAGGGAGAGC	167 bp	305 bp

^1^Score and identity values assessed through alignment between S. italica sequences and S. viridis sequences (TM personal communication). NA indicates non-available data due to missing sequence of S. viridis

Primer3 [33] was used to design the primers, which were constructed to be 20–22 bp in length and to have melting temperatures of approximately 60°C and GC contents ranging between 35–65%. The amplicons varied from 80 to 180 bp in length and were designed to span intronic regions whenever possible. The specificity of the primers was validated both *in silico* and in the RT-PCR profile (S1 Fig). More detailed information about the candidate genes and primers that were used in this study is available in Table 1.

### RNA extraction and cDNA synthesis

A total of five different extraction methods were tested to recover RNA from *S*. *viridis*. These methods included strategies that used phenol extraction, such as the PureLink Plant RNA Reagent and TRIzol (both from Life Technologies), and strategies based on guanidine salt buffer in conjunction with purification columns, such as the Rneasy Mini Kit (Qiagen), InviTrap Spin Plant RNA Mini Kit (Stratec) and SV Total RNA Isolation System (Promega). A detailed manual of RNA handling is available on S1 File. All extractions were performed with an input of 100 mg of grounded tissue, according to manufacturers’ instructions, and representative tissues were taken from the samples for further analysis by qPCR. DNAse treatment was used only in the extraction methods that used purification columns (the Qiagen and Promega methods). The RNA yield produced by each method was quantified using the NanoDrop 2000 Spectrophotometer (Thermo Scientific), and both purity and integrity were assessed via 1% agarose gel electrophoresis with ethidium bromide staining followed by Bioanalyzer2100 (Agilent) verification (S2 Fig). The degree of DNA contamination within each RNA sample was evaluated by RT-PCR (using primers that spanned the length of two exons) and gel electrophoresis analysis (S1 Table; S1 Fig), in addition to analysis of the melting dissociation profile that was obtained during qPCR amplification (S3 Fig).

For cDNA synthesis, an input of 5 μg of total RNA was added to 50 μM of Oligo(dT24 V) primer and 10 mM of each deoxyribonucleoside 5’-triphosphate (dNTPs). The mixture was incubated at 65°C for five minutes and briefly chilled on ice. Following this, 1X First Strand Buffer, 20 mM of dithiothreitol (DTT) and 200 units of SuperScriptIII (Invitrogen) were added to the mixture, to a total final volume of 20 μl. The reaction was incubated at 50°C for 2 hours, after which enzyme inactivation was achieved through a 15 min incubation at 70°C. The cDNA was stored at -20°C.

### Quantitative Real-Time Polymerase Chain Reaction

The amplification reactions were performed in a 7500 Fast Real-Time PCR System (Applied Biosystems) using SYBRGreen to monitor dsDNA synthesis. The reaction mixtures contained 10 μl of diluted cDNA (1:50), 0.2 μM of each primer, 50 μM of each dNTP, 1X PCR Buffer, 3 mM MgCl_2_, 1X SYBRGreen I (Molecular Probes) and 0.25 U of PlatinumTaq RNA Polymerase (Invitrogen), in a total volume of 20 μl. The reaction cycles began with a five minute denaturation step at 94°C, followed by 40 amplification cycles of 15 seconds each at 94°C, 10 seconds at 60°C and a 15 second extension step at 72°C. After each cycle, the fluorescence was measured at 60°C for 35 seconds. The melting curve was produced by a cycle of 95°C for 15 seconds, 60°C for one minute, 95°C for 30 seconds and finally 60°C for 15 seconds. For each tissue, three biological replicates were used, each comprising a pool of at least five individual plants. Additionally, each single qPCR reaction was repeated three times to make technical replicates.

### Analysis of gene expression

Both the efficiency of each experimental set and the *Cq* values that were generated for each qPCR reaction were estimated using the Miner software program [34]. This algorithm employs a three-parameter simple exponential non-linear regression to determine the amplification efficiency that was achieved during each cycle. The average amplification efficiency of each gene was used to calculate the non-normalized expression values, which did not depend on a standard curve. When determining *Cq* values, the Miner software applies the first positive second derivative maximum to find the beginning of each exponential phase (thus the *Cq*).

The non-normalized expression values were calculated by the qBase v1.3.5 software program [35] using the formula *Q* = *E* ^Δ*Cq*^ Q = E^ΔCq^, in which *E* represents the efficiency of gene amplification and Δ*Cq* is the difference between the sample with the lowest expression in the dataset minus the *Cq* value of the sample analyzed. These quantities were then imported into the geNorm [36] and NormFinder [37] analysis tools, which were used according to the directions described in their manuals.

To determine the relative expression of the target genes, *Cq* and efficiency values were submitted to qBase, as described above; however, for the purpose of normalization, two reference genes were selected and their relative expression levels were calculated based on the ΔΔ*Cq* model [38].

## Results

### The relative performance of different RNA extraction strategies

**Fig 2:**
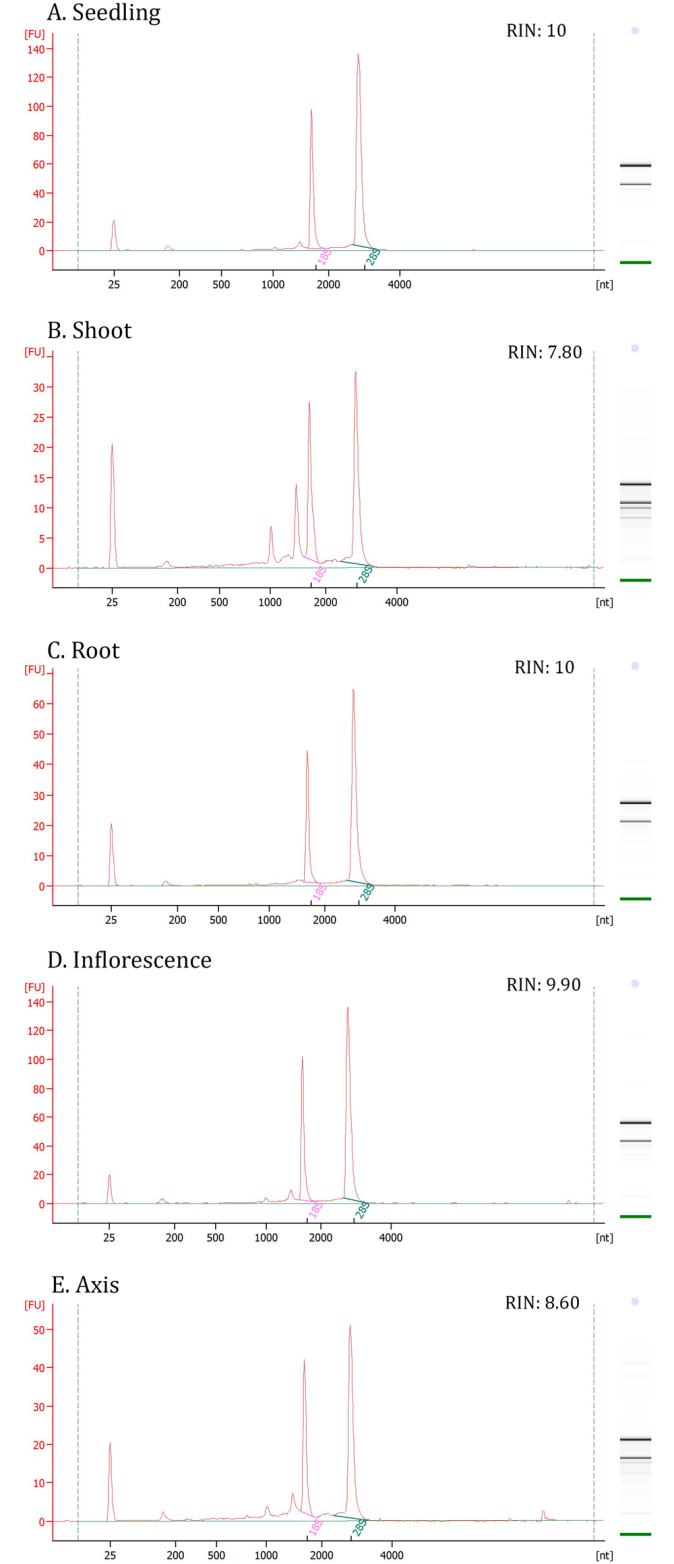
RNA integrity profile. The assessment of the best methodology for RNA extraction for each representative tissue: seedling (A); shoot (B); root (C), inflorescence (D) and axis (E). Electropherograms were obtained using an Agilent 2100 Bioanalyzer. RNA quality is expressed as the RNA integrity number (RIN).

The isolation of high quality RNA is an important factor toward obtaining reproducibility and biological relevance during transcriptional analysis [39]; this motivated us to evaluate the performance of the different methods that can be used to extract *S*. *viridis* RNA. Five different methods were evaluated, and root tissue was found to be the most refractory to RNA extraction; four different methodologies were tested and none could extract significant amounts of RNA from this tissue (S1 Table; S2 Fig). Protocols based on the phenol extraction of RNA also performed poorly with respect to sample purity (S1 Table; S2 Fig). Furthermore, signs of RNA degradation and DNA contamination were observed in samples obtained from both phenol-based and column purifications strategies (S1 Table; S2 Fig). In this study, the SV Total RNA Isolation System (Promega) was found to be the best method for RNA extraction, as it achieved the greatest degree of RNA yield, purity and integrity across a wide arrange of different tissue types (Fig 2).

### Analysis of candidate gene expression profiles and stability

Based on previous studies conducted on major monocot crops [31,32], a total of fifteen candidate genes were chosen for qPCR normalization; the expression profiles of each were analyzed in two different sets of samples. A total of ten traditional housekeeping genes were included in our analysis: three ubiquitin-related genes (Si018608, Si018607 and Si021145), the glycine rich RNA-binding protein (Si025395), actin (Si022372), tubulin (Si035654), elongation factor (Si030042), GAPDH (Si014034), triosephosphate isomerase (Si002651) and the ribosomal protein (Si003209). This collection of genes has been previously used in gene expression analyses of monocots [7,40–43] and dicots [44–48]. The remaining five candidates were amongst the most stable reference genes found during the analysis of maize and rice transcriptomes [31,32]. The first sample set comprised vegetative and reproductive tissues known to participate in the development of *S*. *viridis*, from seedling until mature reproductive phase, and will thus be further referred to as the developmental set. The second group of samples was comprised of different sections from along the C4 leaf blade and will thus be further referred to as the leaf gradient set (Fig 1).

**Fig 3:**
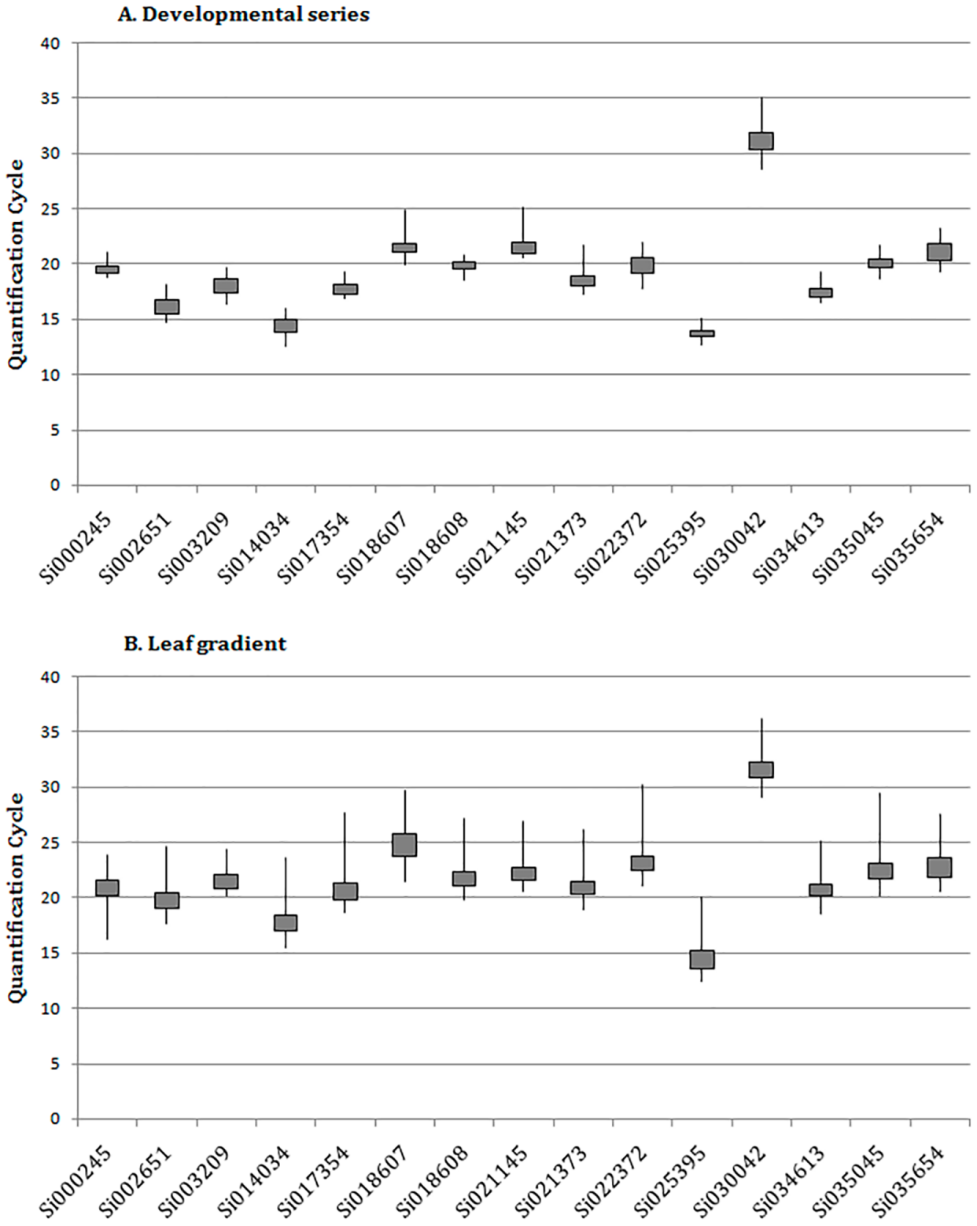
Quantification cycle (*Cq*) boxplot. Graphical representation of *Cq* distribution for each gene analyzed in the developmental (A) and leaf gradient (B) datasets. The line shows the distribution of the maximum and minimum *Cq* values, while the grey box outlines the first and third quartiles.

Regardless of that our primer design was primarily based on the *S*. *italica* genome, the melting dissociation curves that were generated in this study where each representative of the amplification of a single product within each reaction (S3 Fig). Both species share deep similarities [26] that have been previously confirmed by comparison against *S*. *viridis* transcriptome sequencing (Table 1). Sizing the amplicons also confirmed that no genomic DNA contamination was present in the RNA samples. Following qPCR amplification, the *Cq* values and amplification efficiencies were used to assess the expression level of each candidate gene (S2 Table). As shown in Fig 3, the majority of the genes in each of the datasets were found to have an average *Cq* of approximately 20; the lowest *Cq* value was found following amplification of Si025395 (mean *Cq* 13.2) and the highest was found after amplification of Si030042 (mean *Cq* 33.0), both of which were members of the developmental dataset. Because of its low level of expression, Si030042 was removed from further analysis. In this case, the reaction curve did not even reach the maximum of the exponential phase, and any algorithm can provide enough accurate information about the reaction [34].

**Fig 4:**
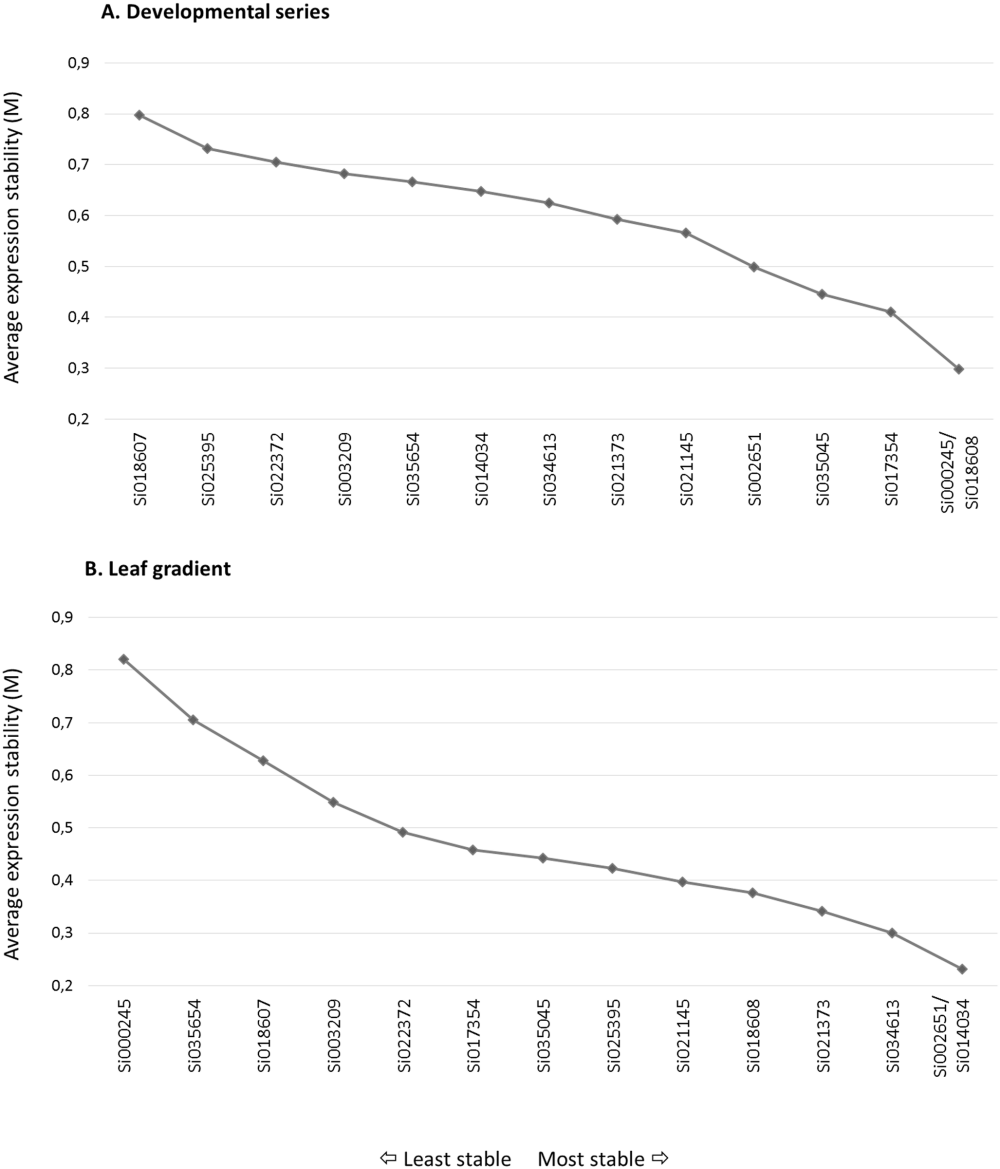
geNorm results for expression stability values (M) and ranking of the candidate reference genes. Average expression stability values (M) of the reference genes measured during geNorm stepwise exclusion of the least stable reference genes. Both the developmental (A) and leaf gradient (B) datasets are shown; lower values of average expression stability, M, indicate more stable expression.

We used two different methods to evaluate the stability of the candidate genes that were used for normalization. The first method, geNorm v3.5, evaluates each control gene to determine its pairwise variation against all other control genes and defines the internal control gene-stability measure, *M*, as the average pairwise variation of a particular gene against all other control genes [36]. Table 2 shows the fourteen genes that were analyzed using geNorm, ranked from the most stable (lower *M*) to the least stable (higher *M*). The most stable gene of the developmental sample set was found to be Si035045, which is a folylpolyglutamate synthase; in the leaf gradient sample set, the cullin Si021373 gene was found to have the lowest *M*. The geNorm algorithm also enabled us to eliminate the worst scoring housekeeping gene, which then allowed us to recalculate new *M* values for the remaining genes. This process was repeated until only two genes remained (Fig 4). As observed in Fig 4a, the Si000245 and Si018608 genes, which encode a protein containing the WD40 domain and an ubiquitin-conjugating enzyme, respectively, were found to be the best pair of genes for the normalization of the developmental set of *S*. *viridis* samples. Likewise, the Si002651 and Si014034 genes, which encode a triosephosphate isomerase and a glyceraldehyde 3-phosphate dehydrogenase (GAPDH), respectively, were found to be the best pair of genes for the normalization of the leaf gradient sample set (Fig 4b).

**Table 2:** Candidate genes ranked by geNorm algorithm according to their average pairwise variation compared with all other genes.

Developmental Set		Leaf Gradient Set	
Ranking	Stability value (M)	Ranking	Stability value (M)
Si035045	0.663	Si021373	0.588
Si018608	0.668	Si018608	0.616
Si000245	0.668	Si021145	0.648
Si017354	0.686	Si034613	0.656
Si002651	0.701	Si014034	0.680
Si014034	0.780	Si025395	0.685
Si021373	0.781	Si002651	0.685
Si034613	0.791	Si035045	0.723
Si021145	0.814	Si017354	0.746
Si035654	0.819	Si022372	0.837
Si003209	0.828	Si003209	0.882
Si022372	0.883	Si018607	1.060
Si025395	0.897	Si035654	1.174
Si018607	1.196	Si000245	1.511

Stability values are listed from the most stable to the least stable.

**Fig 5:**
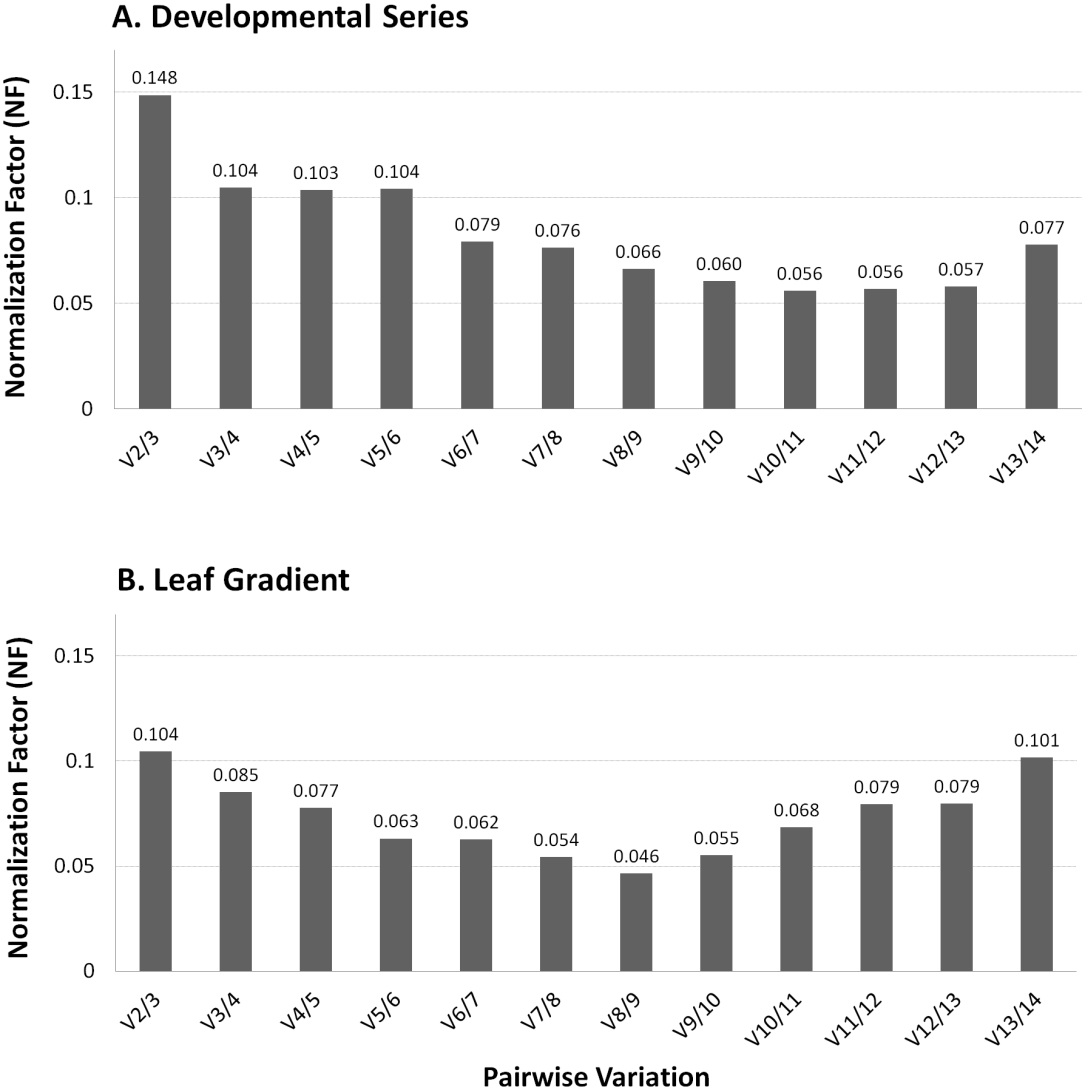
Optimal number of control genes for an accurate normalization. The geNorm pairwise variation *V*_*n*(*n*+1)_ was analyzed between the normalization factors (NF) for both developmental (A) and leaf gradient (B) datasets. All values are below the cutoff of 0.15.

To evaluate expression levels accurately, normalization must be performed using multiple control genes versus using only a single gene [12]. In consideration of this fact, the geNorm algorithm calculates a normalization factor (NF) that is based on the expression level of the best performing housekeeping genes and determines the pairwise variation, *V*_*n*/(*n*+1)_, to measure how the normalization factor is affected by the inclusion of additional reference genes. It is recommended that reference genes are iteratively added to the normalization factor until the last added gene either produces no significant change to this value or until the NF is smaller than 0.15, a cut-off value below which the inclusion of a reference gene is not necessary [36]. The inclusion of two to three reference genes was found to be ideal toward obtaining an accurate normalization of qPCR expression analysis for the developmental and leaf gradient datasets that were included in this study (Fig 5).

The second approach used to evaluate the stability of reference genes was the NormFinder application. This mathematical model splits the dataset to be analyzed into subgroups and estimates the variations in expression between both the intra- and the intergroups to calculate the stability value of a candidate gene. This model-based approach selects the two best genes with minimal combined inter- and intragroup expression variation [37]. In the *S*. *viridis* analysis performed here, the intra group was characterized by each biological replicate, whereas the intergroup comprised the different tissues or sections of the plant. The results from this approach (Table 3) indicated that the best combination of genes for the normalization of the developmental series dataset included the Si034613 and Si035045 genes, which encode a phosphoglucomutase and a folylpolyglutamate synthase, respectively. Likewise, the Si021373 gene, which encodes a member of the cullin family and has been previously reported as the most stable gene, and the Si018608 gene, which encodes an ubiquitin-conjugating enzyme, were noted as the least variable genes in the leaf gradient dataset.

**Table 3:** *S*. *viridis* ranked reference genes and the best combination pair of genes with their stability values calculated by the NormFinder software.

**Developmental Set**		**Leaf Gradient Set**	
**Ranking**	**Stability value**	**Ranking**	**Stability value**
Si017354	0.243	Si021373	0.063
Si035045	0.247	Si018608	0.094
Si000245	0.253	Si021145	0.098
Si018608	0.262	Si034613	0.105
Si002651	0.277	Si002651	0.115
Si021373	0.291	Si017354	0.127
Si021145	0.334	Si014034	0.130
Si034613	0.344	Si035045	0.167
Si014034	0.358	Si025395	0.177
Si035654	0.395	Si022372	0.180
Si022372	0.417	Si003209	0.188
Si003209	0.434	Si035654	0.295
Si025395	0.454	Si018607	0.307
Si018607	0.563	Si000245	0.526
**Best Combination**	**Stability value**	**Best Combination**	**Stability value**
Si034613 and Si035045	0.137	Si018608 and Si021373	0.060

Stability values are listed from the most stable to the least stable.

As expected, geNorm and NormFinder produced different recommendations for the best reference gene because singular statistical inferences are used in these analyses. However, both approaches agreed that Si035045 was among the most stable genes of the developmental series dataset. Similar results were obtained when analyzing the leaf gradient dataset; the cullin family member and ubiquitin-conjugating enzyme were identified as the most stable genes following both of the approaches. The same findings were observed for the geNorm stepwise exclusion result [49,50]. However, both cullin and the ubiquitin-conjugating enzyme may participate in the same pathway [51], and it is not advisable to use reference genes from the same biological process as this may introduce a bias in the normalization process [11,37,47,52]. In summary, the constitutive genes that were used for the normalization of the developmental series dataset (Si034613 and Si035045; Table 4) were chosen based on the results produced by the Normfinder analysis, meeting with the more stable gene in geNorm analysis for this dataset. Whereas the first and the fourth (the next best ranked gene not related to ubiquitin pathway) ranked genes that were indicated in both the geNorm and the Normfinder analyses (Si021373 and Si034613; Table 4) were chosen for normalization of the leaf gradient dataset. A new run that excluded the co-regulated genes was performed for the purpose of confirming the results obtained from the leaf gradient dataset (data not shown).

**Table 4:** Summary of the best normalization pair of genes for each developmental set based on geNorm and NormFinder software programs.

Developmental Set		Leaf Gradient Set	
Gene ID	Biological function	Gene ID	Biological Function
Si034613	Phosphoglucomutase	Si021373	Cullin
Si035045	Folylpolyglutamate synthase	Si034613	Phosphoglucomutase

### Validation of the reference genes

To assess the robustness of the suggested reference genes, the expression levels of well-known, previously characterized genes were analyzed in both sets of data (Table 1). These genes included the putative *S*. *viridis* MADS-box orthologue of AP3/PI, which has been shown to be highly expressed in maize ears and tassels [53]; and PEPC, the enzyme responsible for initial carbon fixation during C4 photosynthesis [19]. The expression patterns of these genes were evaluated in both the developmental and the leaf gradient datasets of *S*. *viridis*.

**Fig 6:**
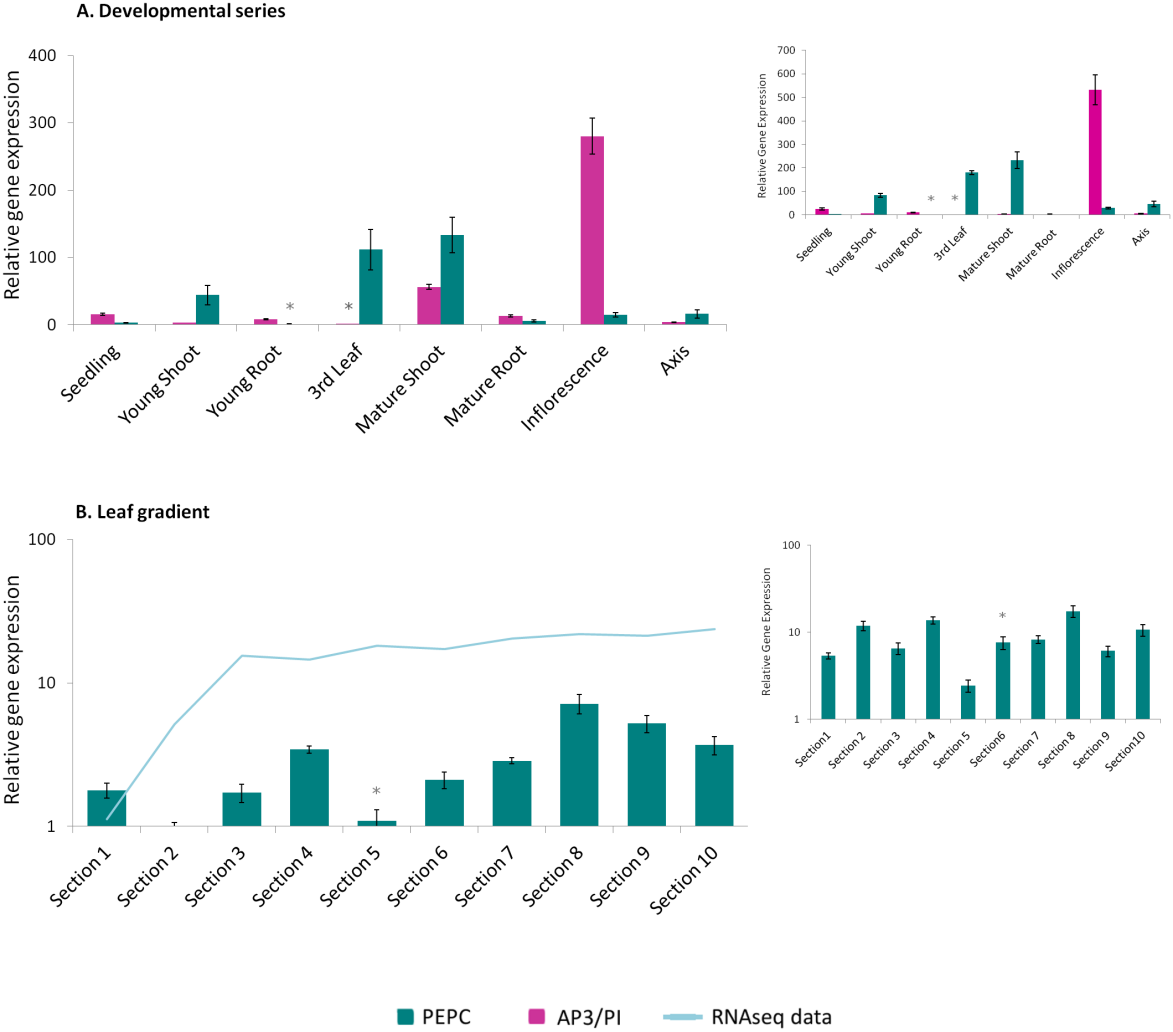
Expression profile of the target genes SvAP3/PI and SvPEPC. The plot corresponding to the expression profile of the MADS-box gene SvAP3/PI (magenta bars) and the enzyme SvPEPC (green bars) responsible for the initial carbon fixation on C4 photosynthesis is shown in (A). The developmental dataset was normalized using the Si034613 and Si035045 genes. It can be observed in (B) that the expression profile of SvPEPC on the leaf gradient was normalized using the gene pair that included Si021373 and Si034613. This result is compared with the transcriptome data (light blue line) for the same gene (personal communication with Todd Mockler). The small plots correspond to the same described target genes normalized with the poorly ranked reference genes: Si018607 and Si025395 for the developmental set and Si000245 and Si018607 for leaf gradient. The reference samples are indicated with an asterix.

The relative expression of AP3/PI in the *S*. *viridis* developmental dataset (Fig 6A) was found to be considerably higher in inflorescence tissue samples compared to other tissues. A lower, but considerable, level of expression was also noticed in samples of mature shoots. These results might also result from sample contamination due to the emergence of immature inflorescences between the leaf sheaths.

Following analysis of the developmental series dataset, the relative expression of PEPC (Fig 6A) was found to be nearly absent in seedlings, roots, inflorescences and flower axes; however, its expression was considerably higher in the photosynthetic tissues that were assessed (third leaf and shoots), as expected. The expression of PEPC was even more evident in tissue samples taken from the third leaves and adult shoots, which are regions of the plant that possess an established C4 metabolism wherein virtually all of the carbon that is fixed by RuBisCO has entered the cycle through a previous fixation step; in this case, through PEPC [21,30]. When evaluating the results produced from the leaf gradient dataset (Fig 6B) it is noticeable that PEPC expression was greatly increased after section three, an observation that was roughly maintained through the leaf tip. It is evident from this pattern that the early developmental stage of the leaf base is in contrast with the fully photosynthetic metabolism of the leaf tip [54]. This finding agrees with previous results from a similar *S*. *viridis* leaf gradient expression analysis study that was conducted via RNA sequencing (personal communication with Todd Mockler; Fig 6B).

For comparison’s sake, the same target genes were normalized with the poorly ranked reference genes for each set. The SvAP3/PI and PEPC were normalized on the developmental set with ubiquitin (Si018607) and glycine-rich protein (Si025395), whereas along the leaf gradient PEPC was normalized with putative leunig protein (Si000245) and ubiquitin (Si018607). These analyses are plotted on the small graphic of Fig 6 and the most striking result is the loss of resolution. Mature root has no expression of either target genes and SvAP3/PI expression, previously observed, in mature shoot due to the presence of upcoming inflorescence is absent. The same process occurs along the leaf gradient, where PEPC expression is nearly constant along the C4 leaf blade.

## Discussion

The accurate normalization of gene expression levels is an absolute prerequisite for obtaining reliable results when using qPCR analysis [36]. Genes encoding transcripts that are involved in basic cellular processes have been the most frequently used for the normalization of qPCR data, as their expression is assumed to be constant amongst different organs and tissues and across a wide range of experimental conditions [15]. However, several reports have suggested limitations to using the so-called housekeeping genes as internal controls because of variations in their expression levels that were observed across different tissues and experimental treatments [15,32,45,55]. In an attempt to standardize qPCR experiments, guidelines were published outlining the minimum information that was required for the publication of data generated by qPCR [MIQE guidelines; 12]. Several studies have since been published with the goal of identifying the normalization genes that represented the best choices for each experimental set that was analyzed [8,31,32,44,45,48,56]. In addition to properly selecting adequate reference genes, several other issues warrant special consideration when trying to avoid the pitfalls of using qPCR to conduct expression analysis; for example, the quality and amount of starting material, the variability of reverse-transcription efficiency and the occurrence of technical errors during PCR setup, all of which may dramatically affect the validity of qPCR outcomes [12].

The first step of an expression analysis experiment, whether conducted by qPCR or high-throughput transcriptome analysis, is crucial to its reliability and reproducibility [12,39]. There are two central issues that impact the successful isolation of RNA. The first is the widespread presence of RNAses in both natural and laboratory settings, as they can rapidly degrade RNA. The second is the presence of primary and secondary metabolites of plants, which can vary dramatically between species and tissues, and include compounds such as phenols and polysaccharides that can interfere with RNA isolation and quality and compromise downstream steps in expression analysis [57]. Just as different plant species produce varying concentrations of compounds that may interfere with a specific RNA extraction method, different extraction methods may also produce different experimental results depending on the species being tested. Especially concerning roots, RNA isolation is generally difficult because of the presence of large quantities of polysaccharides, polyphenols, and other secondary metabolites, in addition to higher fiber and lower RNA content. The high secondary metabolism activity is mainly related to their ability to exude a vast array of compounds into rhizosphere [58]. Taking that into consideration, the first aim of this study was to evaluate the best RNA extraction method for all of the samples that were analyzed. It is important to highlight that using the same methodology for all biological samples is advisable to avoid any bias introduced by the extraction procedure that might affect the expression analysis [59]. Remarkably, in this study a single method was found to be suitable for the recovery of high yield and high quality RNA from all of the samples that were analyzed, thereby increasing the robustness of the expression data that was generated.

Experimental results that are generated using quantitative PCR are critically dependent on the appropriate analysis and interpretation of the outcome of cDNA amplification. Therefore, the workflow used in this paper evaluated important parameters for this analysis. The amplification efficiency, which is described as the fold increases of DNA per cycle and could range from 1 (no amplification) to 2 (complete doubling), was calculated for each gene that was analyzed (S2 Table). It has been previously demonstrated that methods that use reaction-specific PCR efficiencies are less accurate than methods that average the efficiency across all of the reactions of a target gene, due to higher sample specific variability [60]. Another crucial aspect of qPCR data analysis is the estimation of the fluorescence baseline [34,60]. The approach used in the present study included the use of the first positive second derivative maximum to determine the beginning of the exponential phase regardless of the fitting of a straight line to the noise of the first amplification cycles as a baseline, which can especially lead to an erroneous *Cq* estimation when a high concentration of starting template is used [34]. A recent study comparing different qPCR curve analysis methods concluded that there is no single software package that outperforms all others when parameters such as efficiency, bias, precision and increased variation are evaluated, among others. However, the methodology used in this *S*. *viridis* study has been noted as one of the best for estimating the starting amount of target DNA [60].

In agreement with previous results, our study shows that different experimental conditions demand the use of different normalization genes [32,44,45]. Our use of two different algorithms for stability analysis was important for increasing the robustness of our method for choosing the best normalization gene pair for each experimental condition. This strategy agrees with previous studies from our group that were conducted on both cotton and coffee plants [45,48]. We elected Normfinder as the preferential method for selecting the best reference genes for the analysis of the developmental dataset, as it considers both inter- and intra-group variations for the normalization factor. The selected gene pair included the gene encoding folylpolyglutamate synthase (Si035045), an enzyme that catalyzes an important step of the metabolism of tetrahydrofolate (H_4_F-Glu_n_) [61,62]. Folates are present in bacteria, animals and plants, and function in pathways that are involved in controlling cellular viability [63]. In plants, folates are additionally involved in pathways that affect photorespiration and chlorophyll and lignin biosynthesis [63]. The other gene of the selected pair that was used for the normalization of the developmental dataset encodes a phosphoglucomutase (Si034613). This enzyme catalyzes an important traffic point in carbohydrate biosynthesis through the interconversion of glucose 1-phosphate (Glc-1-P) and glucose 6-phosphate (Glc-6-P) [64]. In one direction, the Glc-1-P produced from sucrose catabolism is converted to Glc-6-P, which is the first intermediate in glycolysis. Conversely, the conversion of Glc-6-P to Glc-1-P provides a substrate required for the synthesis of a variety of cells constituents, including cell wall polymers and glycoproteins [64].

When the leaf gradient samples were analyzed, the Normfinder results indicated that the genes encoding the ubiquitin-conjugating enzyme (Si018608) and the cullin (Si021373) were the most stable pair for the normalization of this dataset. However, both of these genes may play roles in the same biological pathway: protein degradation via the proteasome. In this pathway, three ubiquitin-conjugating enzymes (E1, E2 and E3) act in concert to transfer ubiquitin to a substrate protein via a peptide bond, and the resultant complex is targeted for degradation via the proteasome. The cullin identified in our study belongs to a protein family whose members operate as molecular scaffolds to organize the largest class of RING E3 ligases, which are known as cullin-RING ligase complexes (CRLs) [51]. Similar results were found with the gene pair that geNorm analysis indicated was the best; both of the enzymes encoded by this gene pair, triosephosphate isomerase (Si002651) and a glyceraldehyde 3-phosphate dehydrogenase (GAPDH; Si014034), are known to participate in the glycolytic pathway of the Calvin-Benson cycle [49,50,65]. Because co-regulated genes tend to produce very similar expression patterns they tend to be ranked as top choices regardless of their stability [37,47]. Additionally, considering the interest in the carbon-fixation mechanism of C4 photosynthesis, it is not advisable to use reference genes and target genes from the same biological pathway; this is especially true in experiments that analyze control vs. treatment groups, as both target and reference genes tend to be co-regulated. In consideration of this fact, the choice of genes that were used for the normalization of the leaf gradient dataset was based on the genes that were the best ranked but that also had distinct biological pathways. It is noteworthy that both of the genes used were ranked at the same position regardless of whether geNorm or Normfinder analysis was used. This finding indicates the robustness of the overall quality of our validation. In summary, the pair of genes that encoded the *S*. *viridis* cullin family member (Si021373) and the phosphoglucomutase (Si034613) outperformed the other genes that were analyzed in the leaf gradient dataset. Additionally, all genes that were chosen for normalization analysis were ranked to be within the top eight candidates. This result is in agreement with the total number of genes that were analyzed for both experimental datasets [32].

It is important to highlight that classical reference genes, such as actin, tubulin and elongation factor, have shown poor expression stability, which cannot justify their use as normalization genes. This is in accordance with previous studies conducted in wheat and rice, in which novel reference genes outperformed the traditional housekeeping genes in terms of expression stability under all tested conditions [32,55]. A recent study in the closely related *S*. *italica* has also indicated the tubulin gene homologue to be one of the least stable genes for the normalization of samples exposed to saline and hydric stress [8]. In our study, however, genes involved in the traditional pathway of proteasomally-mediated protein degradation were found by both geNorm and Normfinder analysis to be the best ranked combination of leaf development set. In comparison with a similar evaluation in maize, another C4 monocot, the present *S*. *viridis* report is highly consistent. The referred study comprises a wide variety of tissues, developmental stage and stress conditions, has shown cullin and folylpolyglutamate synthase among the top five ranked genes using three different algorithms [31].

When the target genes were normalized with the poor-ranked reference genes, including ubiquitin, it is evident the decrease of sensitivity on the evaluated relative expression. The chosen target genes are highly and specific expressed in certain tissues, making it unlikely to see a shift in their expression, even when poorly normalized. However, on the developmental set, it is clear that SvAP3/PI expression is absent in the mature shoot, an expected result considering the upcoming inflorescences from between the leaf sheaths (see Fig 1). This can be even more drastic, leading to erroneous interpretation, when lower expressed genes are assessed, or when the relative expression difference between tissues are more subtle, as occurs with PEPC expression along the leaf blade. This result illustrates the importance of, before being employed as universal standards, a careful validation of each reference gene candidate in each experimental trial [36,37].

*S*. *viridis* has recently been proposed as a model system for the study of C4 photosynthesis in Panicoid grasses [25] and tools are still under development to strengthen its role as a model plant. The primary aim of this work was to develop a reliable reference of normalization genes for qPCR expression assays. Two sets of data were analyzed in this study. The first dataset was used for a more general exploration of the stability of candidate genes that could be used to normalize the analysis of *S*. *viridis* development and compared different vegetative and reproductive tissues. The second dataset was based on previous reports that characterized the developmental gradient of monocot leaves [30,54]. The leaf was chosen for a deeper expression analysis as it is the major organ responsible for differences in C4 syndrome, and therefore effects regulatory development. To assess the reliability of the chosen reference genes, two target genes with well characterized expression profiles were tested in *S*. *viridis* samples. The first target gene was a MADS-box transcription factor. MADS-box genes have been shown to be involved in the formation of the dehiscence zone, fruit ripening, embryo development and the development of vegetative organs such as roots and leaves; however, these genes are especially well known for their functions in controlling flowering times and meristem and floral organ identity [66]. In maize, several members of this gene class have already been characterized [67]. For our expression analysis in *S*. *viridis*, we chose the orthologue of maize ZmMADS18, a class B gene, due to its high expression levels in ears and tassels [53]. As expected from an AP3/PI gene [66], the *S*. *viridis* orthologue shows an abundant accumulation of transcript within the entire inflorescence.

The gene that encodes PEPC was also analyzed for its expression patterns in both the development and the leaf gradient datasets. In C4 plants this enzyme is responsible for fixing the carbonate into oxalacetic acid, thus initiating the carbon fixation reactions [19]. The expression levels of PEPC that we observed in *S*. *viridis* were consistent with previous studies that have described, through carbon sink-source analysis, the full development of C4 syndrome from the third leaf stage of maize [30]. Additionally, the increased expression of PEPC that was observed towards the third leaf tip is in agreement with recent reports that characterize the leaf gradient as being more developmental at the base, which includes the expression of genes involved in cell division, DNA synthesis and respiration. Conversely, the leaf tip has a photosynthetic metabolism and thus contains higher expression levels of genes that are involved in sucrose synthesis, photosynthesis components and carotenoid biosynthesis [30,54].

## Conclusion

This study was motivated by the importance of the accurate normalization of data that is generated in transcription profiling analyses. To accomplish this goal, select reference genes were evaluated for qPCR normalization, the results of which created a new resource toward the establishment of *S*. *viridis* as a novel Panicoid model. The robustness and reliability of the chosen genes were assessed by evaluating the expression profiles of target genes that have been previously well characterized in other plants, such as *A*. *thaliana* and *Z*. *mays*. This study strongly support the use of Setaria as a model for Panicoid grasses and that the favorability of using this species is further enhanced by its advantageous size, number of seeds, and rapid life cycle.

## Supporting Information

S1 Fig RT-PCR gel profile. Amplified product for each gene pair analyzed using both cDNA and gDNA as template. Reaction was performed with Taq Buffer 1X, MgCl_2_ 2 mM, dNTP 5 uM each, primers 5 mM each forward and reverse and 1 U of Taq DNA Polymerase (Thermo Scientific). As template, 90 ng of gDNA and 1 μl of cDNA was used per reaction. Cycling started at 95°C for 5 minutes, following 32 cycles at 95°C, 58°C and 72°C for 30 seconds each temperature. Final extension was performed at 72°C for 5 minutes. Amplified fragments were loaded on agarose 2% gel and stained with ethidium bromide. No amplification was observed on gDNA for Si025395 in these conditions; the same for Si030042 cDNA due to its low expression.
(TIFF)

S2 Fig Gel profile of RNA extraction. Agarose 1% gel of each RNA extraction methodology for each tissue. Arrows indicate the presence of gDNA contaminant, when visible; the rRNA subunits 28S and 18S and the lower smear indicating degradation.
(TIFF)

S3 Fig Melting curve profile for each reference candidate gene and target gene. SYBRGreen dissociation curve for each of the total of seventeen analyzed genes. The single peak that illustrates the amplification specificity is clearly evident, regardless of the use of the *S*. *italica* genome for primer design.
(TIF)

S1 File Standard Manual Procedure for RNA isolation. Useful rules to obtain a successful RNA extraction.
(DOCX)

S1 Table Comparison of the different RNA extraction methods that were tested on *S*. *viridis* and the evaluation of the yield, purity and integrity of each. ^1^Data corresponds to the extraction of two replicates. Values correspond to mean ± standard deviation. ^1^Missing values substituted by a dash (-) represent samples assessed only once. ^2^NA represents non-assessed data.
(XLSX)

S2 Table Values of efficiency ± standard deviation (SD^1^) for each pair of primer analyzed and the average quantification cycle (Cq) ± SD of the biological replicates for each tissue for the candidates and target genes of *S*. *viridis*. Values were generated by Miner software. NA represents non-assessed data. ^1^The variance observed on standard deviation values, particularly for leaf gradient samples, may be due to sampling method. That is because the boundaries for each section may vary from individuals, leading to higher variance between samples.
(XLSX)
