# Supplementary figures and images for: Validating Internal Control Genes for the Accurate Normalization of qPCR Expression Analysis of the Novel Model Plant Setaria viridis
Source: PLoS One. 2015 Aug 6;10(8):e0135006. doi: 10.1371/journal.pone.0135006 (PMC4527663; doi:10.1371/journal.pone.0135006)

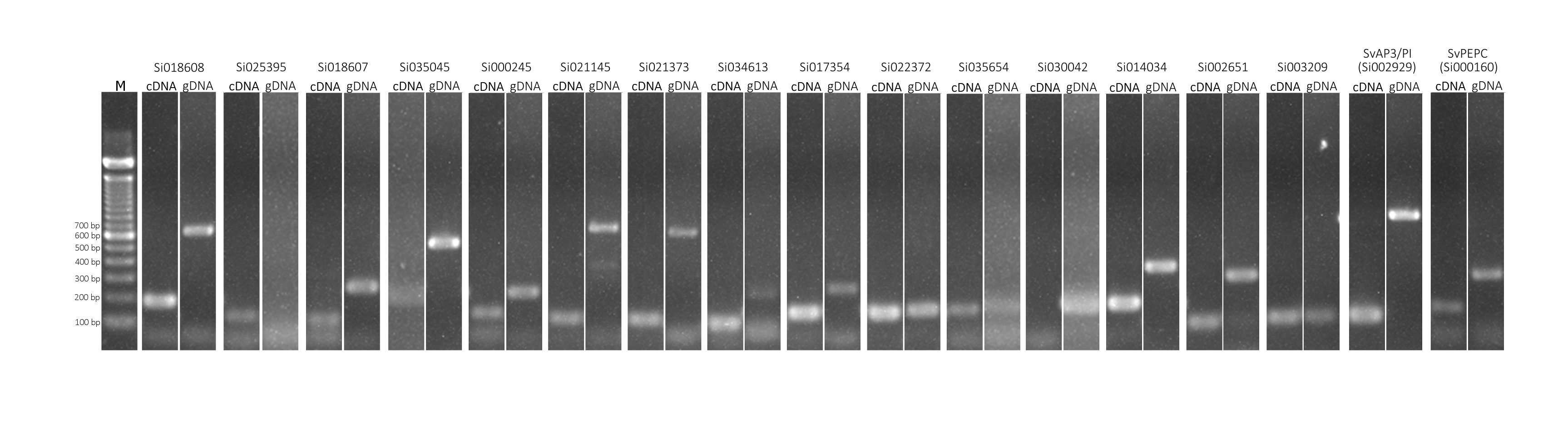

Supplement: S1 Fig — Amplified product for each gene pair analyzed using both cDNA and gDNA as template. Reaction was performed with Taq Buffer 1X, MgCl2 2 mM, dNTP 5 uM each, primers 5 mM each forward and reverse and 1 U of Taq DNA Polymerase (Thermo Scientific). As template, 90 ng of gDNA and 1 μl of cDNA was used per reaction. Cycling started at 95°C for 5 minutes, following 32 cycles at 95°C, 58°C and 72°C for 30 seconds each temperature. Final extension was performed at 72°C for 5 minutes. Amplified fragments were loaded on agarose 2% gel and stained with ethidium bromide. No amplification was observed on gDNA for Si025395 in these conditions; the same for Si030042 cDNA due to its low expression. (TIFF) [file pone.0135006.s001.tiff]

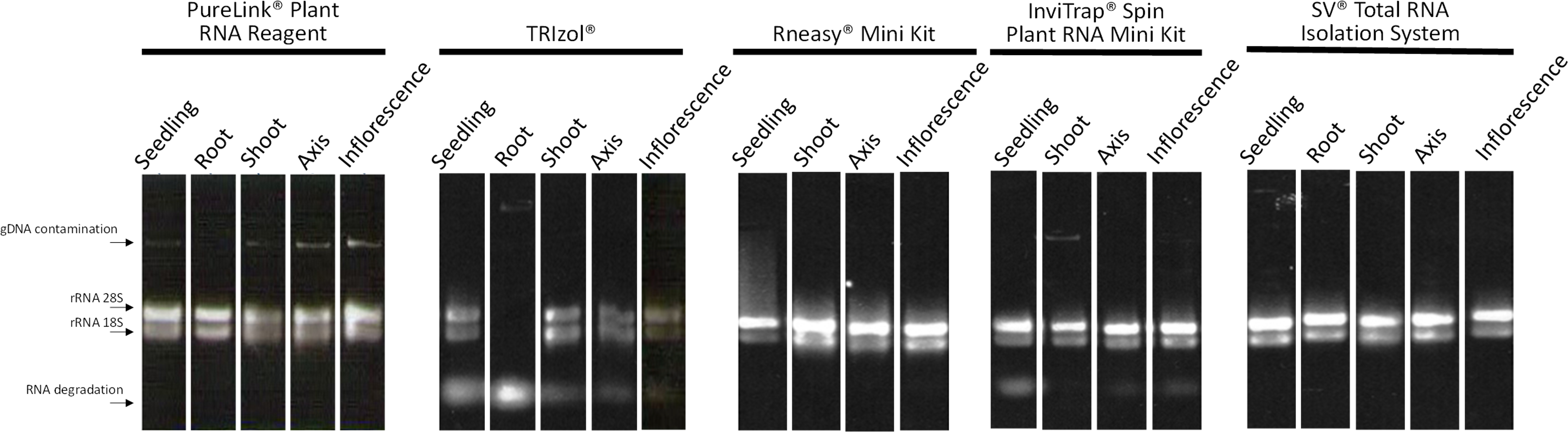

Supplement: S2 Fig — Agarose 1% gel of each RNA extraction methodology for each tissue. Arrows indicate the presence of gDNA contaminant, when visible; the rRNA subunits 28S and 18S and the lower smear indicating degradation. (TIFF) [file pone.0135006.s002.tiff]

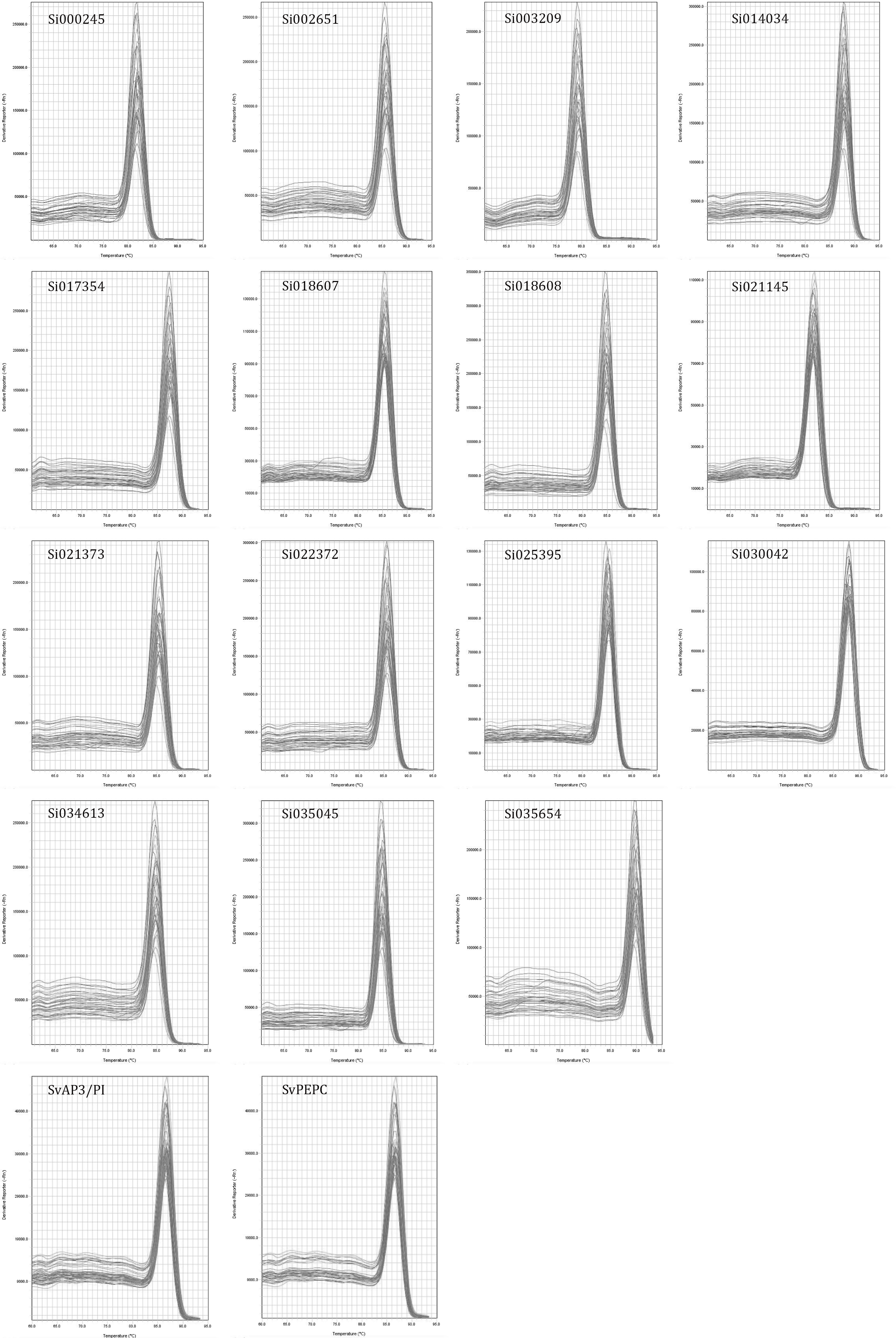

Supplement: S3 Fig — SYBRGreen dissociation curve for each of the total of seventeen analyzed genes. The single peak that illustrates the amplification specificity is clearly evident, regardless of the use of the S. italica genome for primer design. (TIF) [file pone.0135006.s003.tif]
