# Supplementary material for: Validating Internal Control Genes for the Accurate Normalization of qPCR Expression Analysis of the Novel Model Plant Setaria viridis
Source: PLoS One. 2015 Aug 6;10(8):e0135006. doi: 10.1371/journal.pone.0135006 (PMC4527663; doi:10.1371/journal.pone.0135006)
Supplement: S1 File — Useful rules to obtain a successful RNA extraction. (DOCX) [file pone.0135006.s004.docx]

Supporting File 1:

**Standard Manual Procedure for RNA isolation**

Opposite to DNA, RNA is a single stranded polynucleotide molecule that is very susceptible to degradation. In that way, working with RNA requires extra attention due to the chemical instability of the molecule and because the ubiquitous presence of RNAses. Below there is a manual with instructions to ensure a correct manner to handle with RNA.

1. Tips for maintaining a RNAse free environment

- Wear gloves all the time. Avoid touching any contaminated surface and equipment with gloved hands.
- Use sterile, disposable plastic ware whenever possible. Non-disposable plastic ware should be treated with RNAse-inactivating reagents, such as SDS 1% (commercial forms are also available).
- Glassware should be baked at +180˚C for at least 4 hours. Autoclave alone cannot eliminate RNAses from your experiments, because the enzyme can re-naturate after cooling.
- Electrophoresis tanks and combs must be soaked on SDS 1% for 30 minutes and cleaned by wiping.
- Treat water and any liquid solution (except TRIS) that might contact the RNA with DEPC 0.1%. Stir with DEPC overnight and autoclave to hydrolyze any unreacted DEPC.
- Ideally, a special area of the lab should be designated for RNA-work only. Treat surfaces of benches with SDS 1% or commercially available RNAse-inactivating reagents. Rinse the benches with ethanol 70% each time before use to get rid of microorganisms.
- Whenever possible, purchase reagents that are free of RNAses. It is important to be sure that RNA work reagents are separate from general use reagents in the laboratory.

1. Tissue harvesting

- Extract RNA as quickly as possible after obtaining samples. RNA can be degraded by intracellular nucleases. For materials that need handling, it should not be longer than 5 minutes before freezing in liquid N_2_ and stored at -70˚C.
- Select 5-20 plants at the desired developmental stage, this will comprise a pool for one biological replicate. Repeat the procedure as many wanted biological replicates (in this study were used 3 biological replicates).
- If dissection is needed, use a disposable razor blade to harvest the target organ or tissue. Freeze in liquid N_2_ immediately after harvesting.
- Ground each tissue and biological replicate in clean and RNAse-free (see above) pestle and mortar using liquid N_2_.
- Weight 100 mg of grounded tissue and proceed to RNA extraction according to manufacturer`s protocol.
- Store spare grounded tissue, if any, at -80˚C until needed.

1. Handling and storage of RNA

- When working with RNA place all samples on ice. Although DNA is relatively stable at high temperatures, RNA can be easily degraded at temperatures above 65˚C.
- RNA can be dried briefly at +37˚C or in a vaccum centrifuge.
- For long storage or shipping, precipitate RNA in ammonium acetate 5M and store in ethanol 70%, otherwise maintain at -70˚C.

**References**

Working with RNA Manual, Roche Applied Science.

SV® Total RNA Isolation System Technical Manual, Promega.
